# Integrating patient safety education into early medical education utilizing cadaver, sponges, and an inter-professional team

**DOI:** 10.1186/s12909-018-1325-9

**Published:** 2018-09-18

**Authors:** R. Kutaimy, L. Zhang, D. Blok, R. Kelly, N. Kovacevic, M. Levoska, R. Gadivemula, D. Levine

**Affiliations:** Wayne State University, 4201 St. Antoine, 2E UHC, Detroit, MI 48201 USA

**Keywords:** Patient safety, Quality improvement, Curriculum, Anatomy, Medical student, Education, Medical errors, Errors

## Abstract

**Background:**

Introducing patient safety and quality improvement science to medical students is integral to improving healthcare. However, developing and implementing a patient safety curriculum can be challenging in a medical school curriculum that is already densely packed. Our aim was to develop and evaluate the impact of a workshop introducing patient safety and quality improvement science to a large class of first-year medical students.

**Method:**

As a part of an evolving longitudinal patient safety curriculum, an introductory workshop on patient safety was integrated into an anatomy course. A high impact event (a simulated “retained sponge” discovery during an anatomy dissection lab) was used to introduce medical error. The educational session which followed consisted of a presentation by an interprofessional team utilizing the retained sponge as example of an error. Use of safety tools was introduced and quality improvement science was discussed using the evolution of methods to decrease retained foreign objects during surgery. A patient’s story told by a close family member about the personal impact of medical errors was presented. Students then participated in an interactive breakout activity and completed a module on safety. The impact of the workshop was assessed through pre- and post- session tests.

**Results:**

Quantitative and qualitative evaluation reflected a positive effect of the session in improving students’ safety knowledge and attitudes. Students’ mean total knowledge improved from 7.58 to 8.98 (*p* = 0.000). Mean total attitudes score improved from 47.73 to 50.56 (*p* = 0.000). Students’ comments after the workshop reflected increased awareness and appreciation of the importance of addressing medical errors.

**Conclusion:**

A workshop introducing patient safety and quality improvement to first year medical students improved knowledge and attitudes regarding safety and increased awareness of the importance of addressing medical errors in their future careers. Integrating patient safety education into an existing foundational science course is a model for teaching patient safety at other medical schools.

## Background

The significance of medical errors was highlighted by the Institute of Medicine (IOM) in *To Err is Human* [1] *and Crossing the Quality Chasm: A New Health System for the twenty-first Century* [2] and was further addressed with publication of Improving Diagnosis in Health Care [3]. The IOM continues to emphasize enhancing patient safety education and training for all healthcare professionals [4]. The American Association of Medical Colleges (AAMC) calls for educating the next generation of physicians to be fully prepared to recognize vulnerability to error and to participate in patient safety and quality improvement [5]. More recently, the AAMC has identified core entrustable professional activities (EPAs) that trainees should be able to perform on their first day of residency [6]. EPA 13 states that students should be able to “identify system failures and contribute to a culture of safety and improvement” [6].

Implementing a patient safety curriculum focused on these new competencies can be challenging [7]. The traditional medical school curriculum has focused on expanding medical knowledge with an emphasis on basic and clinical sciences with less regard for developing the attitudes, skills, and behaviors needed for delivery of high quality safe care [8].

One obstacle is finding time in a medical school curriculum that is already densely packed [7, 9]. Tsilimingras et al. outlined a variety of additional barriers. Course directors are reluctant to integrate patient safety science into their courses [9]. Many are not yet convinced of the importance of this subject in relation to other subjects for students who have yet to master the foundational sciences such as anatomy, physiology, and biochemistry [7, 9]. Limited clinical time and decreased opportunities for exposure to common patient safety issues are additional concerns [9]. Finally, faculty are uncomfortable teaching outside of their discipline and expertise [9].

A literature review of patient safety education for undergraduate medical students revealed that courses varied greatly in design, content, duration, and evaluation manner. Some were delivered to preclinical students and others were part of clinical electives. Most courses were not formally required in the undergraduate medical curriculum, and only could be selected as an elective [7].

Several medical schools have successfully implemented patient safety training into their clinical clerkships [10–12]. In a curriculum designed for senior medical students in the United Kingdom, Patey et al. evaluated students’ knowledge, attitudes, and behaviors before and one year after completing a 5-h modular curriculum on understanding error in health care. One year later knowledge had improved. Students expressed a high level of satisfaction with the modular format [11]. A 1-day integrated clerkship program for third year medical students at Jefferson Medical College introducing patient safety principles was successful in changing students’ attitudes and beliefs regarding patient safety [12].

Less has been published about preclinical patient safety training. A 10.5-h curriculum including lectures and hands-on training in the computer lab, was delivered to second year medical students at the University of Missouri-Columbia. Pre- and post-testing indicated improvement in students’ attitudes and knowledge. However one year later when students were retested, results revealed that attitudes were not sustained and knowledge related to error analysis declined [13, 14]. This led to development of a booster curriculum for third year students with resultant increases in comfort identifying error [13, 14].

Thompson et al. designed a 10-h curriculum which was delivered to first year medical students at John Hopkins and demonstrated improvement in knowledge and attitudes including future commitment to patient safety [15]. Most other curricula described in the literature have not been delivered to first year students.

Based on this literature, we developed a longitudinal curriculum beginning in the first year of medical school with opportunities for boosting knowledge in every year of education. We had challenges however, identifying time in the curriculum to deliver patient safety science. Inspired by Lynn McNicoll’s use of the anatomy lab and cadavers to teach geriatric principles [16], a preclinical component of the safety curriculum was innovated and embedded into the anatomy course utilizing cadavers as a vehicle to introduce medical error and set the stage for the introductory session. We aimed to determine outcomes of introducing patient safety and quality improvement principles and the value of team work to a large class of early medical students and to determine the short-term impact on knowledge and attitudes related to safety.

## Methods

A session on patient safety was integrated into the anatomy course for first year medical students at Wayne State University. The goals, objectives, mode of delivery (workshop), and assessment were presented to and approved by the Curriculum Committee at the School of Medicine. Expedited approval (124013B3X) was obtained from the Wayne State University Human Investigative Review Board (equivalent to the ethics committee at other institutions) to study the impact and allow for dissemination. The educational session was delivered in October 2014.

### Session development

Students from our medical school’s Institute for Health Care Improvement (IHI) chapter volunteered to be on a subcommittee to develop an introductory session on patient safety and quality improvement as part of a longitudinal curriculum. This group of five second-year medical students met regularly with a faculty advisor. While discussing ways to introduce patient safety, an article describing use of the cadaver lab to teach geriatric principles students was published [16]. Influenced by this article, a decision was made to utilize cadavers and a simulated retained foreign object as an example of a medical error that would be understandable to medical students.

### Procedure

In our medical school dissection of the abdomen is divided into two days. On the first day, students dissect the abdominal wall and open the peritoneum. Two days later, they dissect the contents of the abdomen. Surgical laparotomy sponges were hidden in the abdominal cavity in 16 of 50 cadavers after the first dissection. During second dissection, students discovered the hidden sponges. Students’ reactions and comments were collected by lab instructors and student volunteers. The patient safety educational session was delivered the following week.

The 3-h session began with a 1-h large-group seminar utilizing the retained sponge as an accessible and understandable medical error followed by a discussion of the associated adverse consequences of unintended retained foreign objects and the scope of the problem. The session included a speaker telling the story of his spouse who suffered as a consequence of a medical error and retained surgical object. A presentation by a surgical nurse manager described the role of an interprofessional team including nursing in preventing medical errors. The importance of the team work, speaking up, checklists, debriefs, policies and procedures around sponge counts was discussed. A brief introduction to quality and process improvement describing the evolution of systems to identify retained sponges was presented by a faculty member, although it was not included in pre- and post-session tests.

**Fig. 1:**
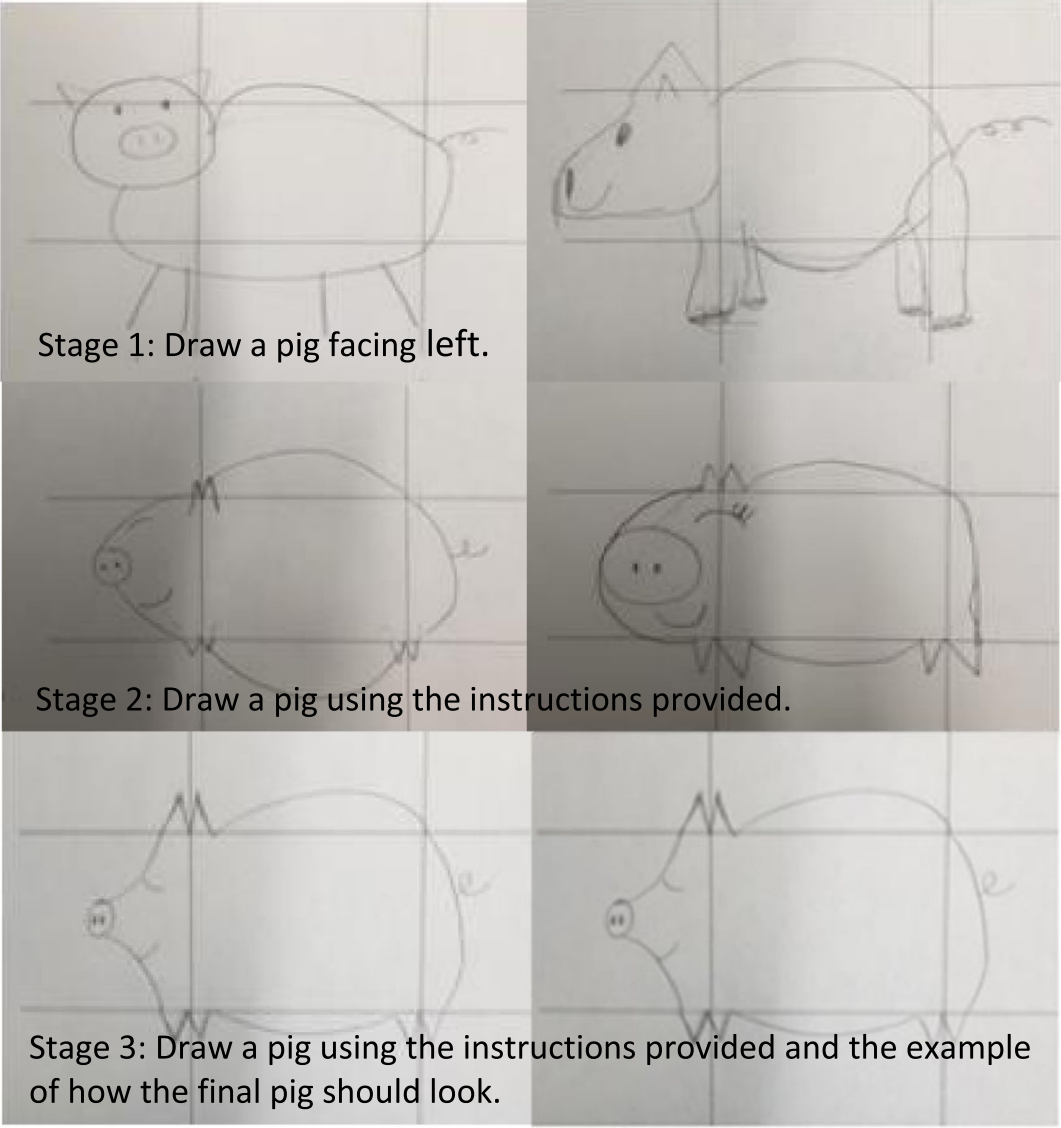
Sample pig drawings from students participating in the workshop

Students then participated in two 45-min breakout sessions which ran simultaneously--a session in a computer lab to complete an introductory patient safety module from the IHI and a facilitated session where groups of 15 students completed the “Standard Pig”. The Standard Pig is a lean simulation exercise which involves drawing a series of pigs with increasingly specific instructions (Fig. 1) [17]. Briefly, students drew pigs freehand. They were then given limited instructions about drawing the pig in profile and facing left. Finally, they were provided with detailed instructions relating to a grid and an example of the end-product and again asked to draw the pig. This exercise illustrates the value of checklists and standard work to ensure high quality outcomes (a uniformly good looking pig).

### Evaluation

A pre-test was conducted to assess students’ attitudes and knowledge related to patient safety; attitudes and knowledge related to process improvement were not tested. Tests of attitudes and knowledge were internally developed based on review of the literature including the IHI modular content [18]. The tests were reviewed for content validity by a faculty member with training in patient safety. Tests were piloted for usability on a group of preclinical students who did not have training in patient safety. Wording was improved and the final test was again piloted.

The pre-test consisted of two parts: the first part evaluated attitudes and included 13 questions. A 5-point Likert scale (1-strongly disagree and 5-strongly agree) was used. On the test of attitudes, reverse scoring was used for the items related to negative attitude (9 items). A composite score was created by summing up the responses of these 13 items (ranging from 3 to 61), with a higher score indicating a positive attitude towards patient safety. These 13 items had an acceptable reliability (Cronbach’s alpha = 0.61).

The second part included 10 multiple choice questions to assess students’ prior knowledge. These 10 items initially had 4 or 5 response choices depending on the question and they were dichotomized into “0 = incorrect” and “1 = correct” for the purpose of data analysis. The sum score was obtained as a composite score (by adding the number of correct answers in response to the 10 items) ranging from 1 to 10, with a higher score indicating a higher level of patient safety knowledge. Because all ten items of knowledge were binary variables, we computed the ordinal version of Cronbach alpha [19]; the value was .62 with this sample. A Cronbach’s alpha of 0.60 in research for the purpose of developing instruments is considered acceptable [20].

At the conclusion of the workshop, students completed an identical post-test to evaluate the immediate impact of the session on attitudes and knowledge. On the post-test, students were additionally asked to identify whether they found a sponge in their cadaver or heard about sponges being found in cadavers prior to the seminar. Students who either found or heard about “retained sponges” were asked to describe their thoughts at the time they found or heard about them. An evaluation was distributed at the end of the session to assess student satisfaction with each component and to allow for feedback and future improvements.

### Analysis of data

Statistical analysis was performed using SPSS 23 for Windows. Normality test was conducted for the total score of attitude and total score of knowledge. Because our data were skewed to the left, Wilcoxon-Mann-Whitneytest was used for continuous variables, and McNemars test was used to binary variables. Mean score (standard deviations, SD), and the proportions of the correct answer of knowledge were presented. *P* values less than 0.05 were considered significant.

## Results

Of 308 students who attended the patient safety training workshop and responded to the Attitude Questionnaire, 281 (91.2%) completed the pre-test, 265 (86.0%) completed the post-test, and 238 (77.3%) completed both pre-, and post-tests. On the Knowledge Questionnaire, 264 (85.7%) completed the pre-test, 260 (84.4%) completed the post-test, and 227 (73.7%) completed both pre-, and post-test.

Table 1 showed that the mean total score of attitudes had significant improvement from pre-test to post-test (47.73 at pre-test vs. 50.56 at post-test, *p* < 0.000). Students’ attitudes towards patient safety statistically significantly improved in nine of 13 items (*p* < 0.01). These nine items included questions 3, 4, 6, 7, 8, 9, 10, 11, and 13. The mean scores of students’ attitudes towards “Making errors while caring for patients is inevitable” (question 1, mean = 2.68 at pre-test vs. mean = 2.79 at post-test, *p* = 0.113), and the attitude towards “Learning how to improve patient safety is an appropriate use of time in medical school” (question 5, mean = 4.03 at pre-test vs. mean = 4.09 at post-test, *p* = 0.343) were slightly improved although these changes did not achieve statistical significance.

**Table 1:** The Mean Score (SD) of Student’s Attitude towards Patient Safety at Pre-test and Post-test (Wilcoxon-Mann-Whitney test) (*N* = 239)

Items	Pre-test, mean (SD)	Post-test, mean (SD)	Difference, mean (SD)	*p*-value
1. Making errors while caring for patients is inevitable (R)	2.68 (1.14)	2.79 (1.21)	0.10 (1.02)^a^	0.120
2. If people paid more attention to their work, medical errors could be avoided (R)	1.97 (0.84)	1.91 (0.79)	−0.05 (0.89)	0.429
3. Patients play an important role in preventing medical errors	3.89 (0.78)	4.09 (0.67)	0.20 (0.76)^b^	0.000
4. Most errors are due to things that physicians can’t do anything about (R)	3.90 (0.66)	4.11 (0.73)	0.21 (0.71)^b^	0.000
5. Learning how to improve patient safety is an appropriate use of time in medical school	4.47 (0.60)	4.53 (0.59)	0.06 (0.62)^a^	0.141
6. If there is no harm to a patient, there is no need to address an error (R)	4.31(0.61)	4.60 (0.60)	0.29 (0.63)^b^	0.000
7. Medical students play an important role in providing patient-centered care	4.11 (0.70)	4.23 (0.66)	0.12 (0.67)^b^	0.006
8. The most important way to reduce medical errors is to have one clear team leader who everybody else follows (R)	3.40 (0.98)	3.84 (0.89)	0.44 (0.88)^b^	0.000
9. Standardizing procedures takes away a clinician’s ability to develop his/her own techniques and eliminates physician creativity (R)	3.45 (0.89)	3.66 (0.91)	0.21 (0.88)^b^	0.001
10. Patient care is provided most efficiently when each team member focuses individually without worrying about what the rest of the team is doing (R)	4.22 (0.74)	4.39 (0.80)	0.17 (0.75)^b^	0.000
11. Most medical errors are because of one provider failing to do his/her job properly (R)	3.43 (0.84)	3.91 (0.91)	0.48 (0.98)^b^	0.000
12. Medical errors used to be a concern, but with modern technology, most providers can make it through their career without committing an error (R)	4.35 (0.69)	4.34 (0.80)	−0.01 (0.79)	0.988
13. Students play a key role in ensuring patient safety	3.89 (0.87)	4.21 (0.63)	0.33 (0.79)^b^	0.000
Total score of attitude scale (score range: 3–61)	47.73 (5.10)	50.56 (4.46)	2.83 (4.75)	0.000

*SD* Standard Deviation

^a^Item with improvement on attitude

^b^Item with statistical significance in improvement

Results in Table 2 indicated that the mean total score of knowledge related to patient safety improved in the post-workshop test compared to the pre-workshop (8.98 at post-test vs. 7.58 at pre-test, *p* < 0.001). In addition, students’ patient safety knowledge improved individually in most items (8 of 10) showing statistical significance (questions 1–7, 10). Students’ ability to recognize the type of medical error (question 3 and 4) improved significantly as means in the two medical errors questions increased by 37.6% and 29.6%, respectively (*p* < 0.000) [18]. One question related to effective systems and interprofessional teams (question 8) did not show statistical significance in improvements from pre-test to post-test, although the percentage of correct answers improved from 97.1% at pre-test to 99.1% at post-test. On another question related to the point of protocols (question 9), students’ knowledge increased from 89% at pre-test to 92% at post-test but again did not achieve statistical significance.

**Table 2:** The percentage of correct answer of Student’s Patient Safety Knowledge at Pre-test and Post-test (McNemars test test)

Items	Pre-test, n (%)	Post-test, n (%)	Difference, n (%)	*p*-value
1. Which of the following is most likely to decrease the risk of medical errors?				
*b. Examine how the system is setting providers up to make errors and try to fix it*				
*(Yes = 1, Else = 0)*	162 (72.3)	185 (82.6)	23 (0.10)	0.001
2. Which of the following is true regarding medical errors?				
*c. An error that doesn’t result in any harm is an opportunity to find a hole*				
*in the system and prevent future harm (Yes = 1, Else = 0)*	210 (94.2)	221 (97.8)	11 (0.05)	0.039
3. A student incorrectly records a patient’s drug allergies, this is classified as what type of medical error?				
*a. Slip (Yes = 1, Else = 0)*	53 (24.0)	138 (61.1)	85 (0.38)	0.000
4. A physician forgets to order a patient’s medications, this is classified as what type of medical error?				
*b. Lapse (Yes = 1, Else = 0)*	145 (65.6)	212 (93.8)	67 (0.30)	0.000
5. A physician misreads a radiograph resulting in a misdiagnosis, this is classified as what type of medical error?				
*c. Mistake (Yes = 1, Else = 0)*	170 (78.0)	198 (87.2)	28 (0.12)	0.006
6. A surgeon rushes the surgical team to start surgery, ignoring the charge nurse’s wishes to perform a timeout, this is classified as what type of medical error?				
*d. Violation (Yes = 1, Else = 0)*	192 (87.7)	223 (98.2)	31 (0.14)	0.000
7. Which individuals are likely affected by a surgical error?				
*e. All of the answers (*The patient and his family; The physician in charge of the surgery; The second-year resident assisting the physician; The nurse on the team) *(Yes = 1, Else = 0)*	213 (95.9)	224 (98.7)	11 (0.05)	0.031
8. Effective systems focused on ensuring safety and preventing errors require:				
*d. Collaboration of the interprofessional team with the patient and family (Yes = 1, Else = 0)*	213 (97.3)	223 (99.1)	10 (0.04)	0.125
9. What is the point of a protocol?				
*a. To standardize the delivery of care and help providers perform optimally*				
*(Yes = 1, Else = 0)*	191 (88.8)	207 (92.0)	16 (0.07)	0.265
10. Most patient harm is the result of:				
*c. A series of system errors (Yes = 1, Else = 0)*	172 (79.3)	208 (92.0)	36 (0.16)	0.000
Total score of knowledge scale (score range:1–10), mean (SD)	7.58 (1.82)	8.98 (1.22)	1.40 (1.89)	0.000

### Student comments

Fifty-eight students were assigned to cadavers with sponges while 214 were not; 36 students (62.1%) found sponges, 21 students (36.2%) did not find the sponge, 1 student (1.7%) did not answer the question. Thirty-one additional students heard about the sponges.

Comments from students who saw or heard about the sponge in the cadaver were reviewed. Five themes related to patient safety and quality emerged. Students recognized a retained sponge as a medical error and presumed that an error had occurred and it could have caused a serious adverse event such as sepsis or death. Many students were very surprised that this could still happen despite the advanced care and large number of staff in surgeries. Assuming the sponges were left after surgery, students identified poor surgical quality and commented that such an error can lower patients’ confidence in surgeries. Student reactions reflected their emotional discomfort (disappointment, fear, sadness, and disgust) after finding or hearing about the sponges. Thinking that the sponges were medical errors and might have caused patient deaths increased the students’ awareness of the importance of addressing medical errors in their future careers.

Students were highly satisfied with the seminar with 94% rating the day as good to excellent. The speaker’s presentations were well received. Student most appreciated the nurse manger’s presentation with 99% rating as good to excellent. Students similarly highly rated the presentation by the husband of a patient who experienced a medical error with 92% rating as good to excellent. 308 students completed the pig drawing session. Students enjoyed the session rating the activity as very good to excellent by 89%. The modules were liked by more than 70%, but students noted that 45 min was inadequate time to complete the module.

## Discussion

We were able to introduce patient safety principles to early first year medical students utilizing the anatomy lab, cadavers and a limited amount of time in the anatomy course. The delivery of patient safety principles by diverse health care providers introduced students to the values of team work and the culture of safety. Pre-and post-test assessment demonstrated significant improvement in students’ overall safety knowledge and attitudes. The session was very well received.

We believe the accessibility of the retained sponge experience and emotional discomfort prepared students for this educational session. Emotion is now considered an essential component of learning science [21, 22]. Students’ comments indicated heightened awareness of the need to be careful in their future practice and increased willingness to change behavior to be more aware of errors and work to prevent errors. The finding of sponges in cadavers without incisional scars was believable. Preserved cadavers have decreased volume and creases that may be misinterpreted as surgical scars (personal observation by DL). Believability was enhanced as students’ focus was directed on the dissection and not on the surface anatomy.

There are different ways to accomplish patient safety and quality improvement education. Taking advantage of existing opportunities and resources (e.g. the cadaver lab) we integrated an early educational experience in patient safety at our school. Negotiating adding time in the curriculum required support by the course director of anatomy and the curriculum committee and was necessary to deliver the workshop. Due to our large class size (300 students per year) using students with an interest in patient safety to help develop and deliver the curriculum was also crucial to success. Students not only planted the sponges in the cadavers they facilitated 16 of 20 Standard Pig drawing sessions.

Introduction of a curriculum in patient safety through use of an introductory session delivered during anatomy should be feasible at other institutions. The paradigm of working within the existing curricular structure to identify a course, available time, and resources is a model for other schools. Finally, use of student patient safety champions provided an enthusiastic resource for curricular development and delivery. This experience is part of an evolving longitudinal patient safety curriculum. Our goal was to engage students and begin education early.

## Conclusion

A session to deliver patient safety concepts to first year medical students integrated into an existing course resulted in improvement in overall patient safety knowledge and attitudes. Students’ reactions to the discovery of the sponges reflected their ability to recognize medical errors and their consequences. Students responded emotionally to these errors and expressed their surprise and discomfort which led to an increasing awareness that more care should be applied throughout their career.

We provide a model for teaching medical students’ patient safety principles by implementing into existing curricula in the basic science years to increase the students’ knowledge, skills and attitudes related to patient safety and quality improvement.

## Appendix 1

**Table 3:** Items of attitude scale

Items	Mean (S.D.)	Corrected item-total correlation
1. Making errors while caring for patients is inevitable. (R)	2.69 (1.13)	0.17
2. If people paid more attention to their work, medical errors could be avoided. (R)	1.97 (0.86)	−0.18
3. Patients play an important role in preventing medical errors.	3.87 (0.79)	0.08
4. Most errors are due to things that physicians can’t do anything about. (R)	3.90 (0.70)	0.37
5. Learning how to improve patient safety is an appropriate use of time in medical school.	4.45 (0.63)	0.28
6. If there is no harm to a patient, there is no need to address an error. (R)	4.29 (0.66)	0.44
7. Medical students play an important role in providing patient-centered care.	4.10 (0.71)	0.43
8. The most important way to reduce medical errors is to have one clear team leader who everybody else follows. (R)	3.39 (0.96)	0.17
9. Standardizing procedures takes away a clinician’s ability to develop his/her own techniques and eliminates physician creativity. (R)	3.47 (0.93)	0.35
10. Patient care is provided most efficiently when each team member focuses individually without worrying about what the rest of the team is doing. (R)	4.21 (0.75)	0.29
11. Most medical errors are because of one provider failing to do his/her job properly. (R)	3.41 (0.86)	0.23
12. Medical errors used to be a concern, but with modern technology, most providers can make it through their career without committing an error. (R)	4.33 (0.72)	0.20
13. Students play a key role in ensuring patient safety.	3.91 (0.85)	0.44
Attitude scale score, mean (SD), Cronbach’s alpha (score range: 3–61)	47.64 (5.18)	0.61

*(R)* Reversed items

## Appendix 2

**Table 4:** Items of knowledge scale

Items	Mean (S.D.)	Corrected item-total correlation
1. Which of the following is most likely to decrease the risk of medical errors?		
a. Accept them as an inevitable outcome of patient care and focus very hard on avoiding them	0.71 (0.46)	0.12
***b. Examine how the system is setting providers up to make errors and try to fix it***		
c. Make it clear to providers that errors will not be tolerated, and discipline providers who make errors leading to patient harm		
d. Since trainees are responsible for most errors, increase the length of training to ensure everyone is competent		
2. Which of the following is true regarding medical errors?		
a. If no lasting harm is done to the patient, it’s not really an error and doesn’t need to be addressed	0.94 (0.23)	0.14
b. Patients are unable to tell when an error occurs, so their opinion should be ignored		
***c. An error that doesn’t result in any harm is an opportunity to find a hole in the system and prevent future harm***		
d. After an error occurs, the hospital should avoid telling anyone, and solve it on their own without involving the patient		
e. Ever since the IOM report “To Err is Human”, the number of medical errors has decreased and it’s no longer a problem		
3. A student incorrectly records a patient’s drug allergies, this is classified as what type of medical error?		
***a. Slip***	0.24 (0.43)	0.28
b. Lapse		
c. Mistake		
d. Violation		
4. A physician forgets to order a patient’s medications, this is classified as what type of medical error?		
a. Slip	0.64 (0.48)	0.34
***b. Lapse***		
c. Mistake		
d. Violation		
5. A physician misreads a radiograph resulting in a misdiagnosis, this is classified as what type of medical error?		
a. Slip	0.77 (0.42)	0.20
b. Lapse		
***c. Mistake***		
d. Violation		
6. A surgeon rushes the surgical team to start surgery, ignoring the charge nurse’s wishes to perform a timeout, this is classified as what type of medical error?		
a. Slip	0.88 (0.33)	0.36
b. Lapse		
c. Mistake		
***d. Violation***		
7. Which individuals are likely affected by a surgical error?		
a. The patient and his family	0.94 (0.24)	0.25
b. The physician in charge of the surgery		
c. The second year resident assisting the physician		
d. The nurse on the team		
***e. All of the above***		
8. Effective systems focused on ensuring safety and preventing errors require:		
a. Collaboration among physicians	0.97 (0.17)	0.33
b. Collaboration among nurses		
c. Collaboration among patients and families		
***d. Collaboration of the interprofessional team with the patient and family***		
9. What is the point of a protocol?		
a. To standardize the delivery of care and help providers perform optimally	0.87 (0.34)	0.19
b. To standardize the delivery of care and protect providers from lawsuit if an error occurs		
c. To standardize the delivery of care so that patients know what to expect when they go to the doctor		
d. To standardize the delivery of care and prevent providers from developing their own techniques		
10. Most patient harm is the result of:		
a. A bad decision from one individual on the team	0.76 (0.43)	0.17
b. The bad performance of a physician		
***c. A series of system errors***		
d. Lack of time		
e. The complexity of the procedure		
Knowledge scale score, mean (SD), Cronbach’s alpha (score range: 1–10)	7.53 (1.83)	0.62

Bold, Italic, and underline items are correct answers

## Abbreviations

AAMC: American Association of Medical Colleges
DL: Diane Levine
EPAs: Entrustable professional activities
IHI: Institute for Health Care Improvement
IOM: The Institute of Medicine
SD: Standard deviations
SPSS: Statistical Package for the Social Sciences
